# Preliminary validation of the PRImary care facility Management Evaluation tool (PRIME-Tool), a national facility management survey implemented in Ghana

**DOI:** 10.1186/s12913-019-4768-8

**Published:** 2019-12-05

**Authors:** Jhanna Uy, Erlyn K. Macarayan, Hannah L. Ratcliffe, Kate Miller, Easmon Otupiri, John Koku Awoonor-Williams, Lisa R. Hirschhorn, Stuart R. Lipsitz, Dan Schwarz, Asaf Bitton

**Affiliations:** 1 Ariadne Labs, Brigham and Women’s Hospital & Harvard T.H. Chan School of Public Health, 3rd Floor East, 401 Park Drive, Boston MA, Boston, MA 02215 USA; 2 Department of Global Health and Population, Harvard T.H. Chan School of Public Health, Boston, MA USA; 3 Kwame Nkrumah University of Science and Technology, Kumasi, Ghana; 4 Policy Planning Monitoring and Evaluation Division, Ghana Health Service PMB, Accra, Ghana; 5 Feinberg School of Medicine, Northwestern University, Chicago, IL USA; 6 Center for Surgery and Public Health, Brigham and Women’s Hospital, Harvard Medical School, Boston, MA USA; 7 Division of Global Health Equity, Brigham and Women’s Hospital, Boston, MA USA; 8 Center for Primary Care, Harvard Medical School, Boston, MA USA; 9 Department of Health Care Policy, Harvard Medical School, Boston, MA USA; 10 Division of General Medicine, Brigham and Women’s Hospital, Boston, MA USA

**Keywords:** Primary health care, Facility management, Measurement, Survey, Exploratory factor analysis, Ghana

## Abstract

**Background:**

The management quality of healthcare facilities has consistently been linked to facility performance, but available tools to measure management are costly to implement, often hospital-specific, not designed for low- and middle-income countries (LMICs), nor widely deployed. We addressed this gap by developing the PRImary care facility Management Evaluation Tool (PRIME-Tool), a primary health care facility management survey for integration into routine national surveys in LMICs. We present an analysis of the tool’s psychometric properties and suggest directions for future improvements.

**Methods:**

The PRIME-Tool assesses performance in five core management domains: Target setting, Operations, Human resources, Monitoring, and Community engagement. We evaluated two versions of the PRIME-Tool. We surveyed 142 primary health care (PHC) facilities in Ghana in 2016 using the first version (27 items) and 148 facilities in 2017 using the second version (34 items). We calculated floor and ceiling effects for each item and conducted exploratory factor analyses to examine the factor structure for each year and version of the tool. We developed a revised management framework and PRIME-tool as informed by these exploratory results, further review of management theory literature, and co-author consensus.

**Results:**

The majority (17 items in 2016, 23 items in 2017) of PRIME-Tool items exhibited ceiling effects, but only three (2 items in 2016, 3 items in 2017) showed floor effects. Solutions suggested by factor analyses did not fully fit our initial hypothesized management domains. We found five groupings of items that consistently loaded together across each analysis and named these revised domains as Supportive supervision and target setting, Active monitoring and review, Community engagement, Client feedback for improvement, and Operations and financing.

**Conclusion:**

The revised version of the PRIME-Tool captures a range of important and actionable information on the management of PHC facilities in LMIC contexts. We recommend its use by other investigators and practitioners to further validate its utility in PHC settings. We will continue to refine the PRIME-Tool to arrive at a parsimonious tool for tracking PHC facility management quality. Better understanding the functional components of PHC facility management can help policymakers and frontline managers drive evidence-based improvements in performance.

## Background

Available literature from low- and middle-income countries (LMICs) and recent work in Ghana show that better management of primary health care (PHC) facilities and hospitals is associated with better facility performance and patient experience outcomes [1–6]. In developed countries, good facility management has been consistently linked to improved service delivery quality and clinical outcomes [7–12]. With the landmark Astana Declaration of October 2018, the global community has reaffirmed primary health care as a foundational strategy to achieve universal health coverage and equitable health for all [13]. As such, better management of PHC facilities will be a critical lever in improving the quality and performance of PHC systems in developing countries.

However, the management of PHC facilities in LMICs is still under-studied and poorly understood [4, 5]. As most evidence comes from hospital settings in high-income countries, valid and reliable tools to measure PHC facility management in LMICs are lacking. Past studies on healthcare facility management have relied on methods that are difficult and costly to implement routinely in developing countries, such as case studies, 360° reviews of individual staff and manager performance, and qualitative interviews [1, 3, 10, 14]. Meanwhile, existing evaluation tools for PHC facilities often focus on measuring key inputs or functions of primary health care such as first contact access and continuity, with almost no focus on measuring core *management* functions; moreover, they are also commonly unwieldy in their length or implementation [15–22].

The World Management Survey (WMS) is a well-validated management survey tool, but in the health care realm has been applied primarily to hospitals in developed countries [23]. The WMS cannot be directly applied to the measurement of management performance of PHC facilities in LMICs for two main reasons. First, the methods employed by the WMS involving extensive qualitative interviews are time consuming, expensive, and difficult to scale in resource-constrained settings [11]. Second, the context of PHC facilities in LMICs is fundamentally different from large hospitals in developed countries [1, 5, 24]. Consequently, management practices will manifest differently and therefore must be assessed differently. For example, effective human resource management - though based on the same principles - will look very different at a tertiary hospital employing hundreds of specialized staff than in a small PHC facility with only a few health workers.

Without good measurement, health officials and front-line managers are not aware of the performance of their PHC facilities, and much less able to address gaps and problems in facility management functions. The absence of strong measurement impedes the ability of LMICs to deliver on their promise of affordable and essential quality PHC for all. To address this measurement gap, we developed a new tool to measure PHC facility management, the PRImary care facility Management Evaluation tool (PRIME-Tool), which LMICs can use for nationwide monitoring.

The first version of the PRIME-Tool was developed by the Primary Health Care Performance Initiative (PHCPI) [25] in 2015 based on the conceptual domains of the WMS, but geared toward PHC facilities in LMICs. The PRIME-Tool was first fielded in Ghana in 2016 [6], and fielded again in Ghana in 2017 with seven new items. In this analysis, we used data from both years to assess the measurement properties of the PRIME-Tool, specifically its response variability across years, floor and ceiling effects, and factor structure. This is an intermediate step in the development of the tool, and we expect to refine the PRIME-Tool moving forward to improve its measurement properties.

## Methods

### Structure of the PRIME-Tool

The PRIME-Tool was developed to measure management processes in PHC facilities in developing countries. It incorporates four core management domains adopted from the WMS - Target setting, Operations, Human resources, and Monitoring. We added a domain for Community engagement, which is essential to PHC management. The individual items of the PRIME-Tool were constructed to address all five of these domains from a PHC perspective. The original version as fielded in 2016 included 27 items: three items measuring Target setting, six measuring Operations, four measuring Human resources, eight measuring Monitoring, and six measuring Community engagement (Table 2). Based on enumerator and respondent feedback from the first fielding of the PRIME-Tool [6], we added seven items to capture additional important management activities: four new items in Target setting, two new items in Human resources, and one new item in Monitoring. A detailed discussion of the development of the PRIME-Tool is available in Additional file 1: Instrument development.

The 2017 version of the PRIME-Tool has a total of 34 items: 21 binary items (yes/no questions), 12 ordinal items (Likert scales) and one continuous item (proportion of time the PHC facility head spent on managerial activities). Responses on each item were re-scaled from 0 to 1, with 1 representing better management (see Additional file 2: The PRIME-Tool for the scores assigned to item choices). Scaling for each item was pre-determined and these scores were not visible to enumerators and respondents in the field survey used. A facility is surveyed once, and facility scores for items are then determined by analysts using this scoring key during data processing.

### Fielding the PRIME-Tool in Ghana

Ghana is a lower middle income country with a GDP per capita of USD 1641.50 and a population of 28.83 million in 2017 [26]. It is divided into 10 administrative regions and 216 districts which are further subdivided into approximately 38,000 enumeration areas (see Additional file 3 for a visualization) [27]. Healthcare is provided by both the public and private sector. Primary health care in Ghana is delivered through Community-Based Health Planning and Services (CHPS) compounds, health centers and clinics, and district/primary hospitals [28].

Our sample of PHC facilities represents the facilities used by and accessible to a nationally representative sample of women of reproductive age surveyed by the Performance Monitoring and Accountability 2020 (PMA 2020) program [29]. Briefly, PMA 2020 assembled a probability sample of women from 100 randomly chosen enumeration areas (EAs) across Ghana using multistage cluster sampling stratified by urban-rural EAs [29]. These EAs chosen for the PMA 2020 survey were the same EAs used to sample health facilities. For the first fielding of the PRIME-Tool in 2016, all public facilities that serve the EA and three randomly chosen private facilities from a list of all private facilities within EA boundaries were selected. For 2017, the same EAs and public facilities were surveyed, but for each EA, three private facilities in each EA were again randomly chosen.

A trained enumerator interviewed one primary respondent from each facility from September to December 2016, and November 2017 to February 2018. Respondents included medical directors and superintendents, directors of nursing, or nursing matrons for hospitals and health centers, and midwives or community health nurses for CHPS. For private facilities, respondents were: facility owners, managing partners, facility administrators, or highest-ranking doctors. If necessary, enumerators were referred to other staff for items the primary respondents were not knowledgeable about. All facility surveys were administered in English.

### Statistical analysis

To provide descriptive context about the survey sample, we first calculated distributions of facility characteristics from each year: region, managing authority, type of facility, number of beds, and participation in the national health insurance system.

Second, to explore variation in raw responses across years, we calculated the mean scores for each indicator in each year. For binary variables, the mean is the proportion who answered “Yes” to the item. For ordinal and continuous variables, standard deviations were also calculated. We did not calculate the statistical significance of differences across years because our purpose was to understand the functioning of the PRIME-Tool, not to make inferences about changes in the underlying conditions in Ghana. Floor and ceiling effects were computed as the proportion of facilities that scored the lowest possible score (0) or highest possible score (1) on each item, respectively [30]. Thresholds for potentially problematic floor and ceiling effects were set to 80% for binary variables and 15% for ordinal variables. A threshold of 15% is commonly used in evaluating floor and ceiling effects for ordinal variables in literature [31]. However, we could not find references that specified a threshold for binary variables. Since the variance of a binary variable is lowest when the proportion of responses for a choice is near 100%, we decided that a threshold of 80% was reasonable to adopt. This follows the principle that thresholds should evaluate the ability of the PRIME-Tool to produce good variation in scores or distinguish facility performance at both ends of the scale.

Last, we evaluated the PRIME-Tool using Exploratory Factor Analysis (EFA) to see if the data would reveal a factor structure similar to our original conception of management for primary health care facilities. The initial development of the PRIME-Tool was based on five conceptual domains guided by the WMS, implying a hypothesized five-factor structure. Often, Confirmatory Factor Analysis (CFA) is used to test whether a dataset fits a strong and pre-existing hypothesized factor structure. However, theory on what to evaluate and how to evaluate management practices for primary care facilities in developing countries is still in its early stages [22]. Thus, we chose to use Exploratory Factor Analysis (EFA) because it does not impose constraints on the data [32]. This gives us greater flexibility in discovering underlying factor structures that better fit the items and which may lead to revisions that could improve the tool’s conceptual framework.

Prior to the EFA, we excluded some items beyond the control of facility management. For example, whether a facility was “accountable for health outcomes of a defined group of people” depends not on local management but the public health care system, which defines catchment areas for facilities. Six items were removed for this reason: measures coverage of key population indicators (item #1), reports accountability for health outcomes of a group of people (item #3), health worker present in the facility 24 h a day (item #9), open every day (item #10), user fees displayed (item #12), and tracks common conditions (item #25). Removing these seven left 20 items in the 2016 data and 27 in the 2017 data for the EFA analysis. An item on having a hand washing area with soap and water (item #8) was removed due to significant ceiling effects. Moreover, the item was not a core management activity, but rather a potential result of facility management.

We explored the factor structure for both years separately to gain insight on its stability from year to year and to examine how the factor structure would change with the new questions included in the 2017 version of the tool. The first model, EFA_1_, used the 2016 PRIME-Tool data with 20 items. To make the year-to-year data comparable, we estimated EFA_2_ using the 2017 data and only the 20 items that were included in 2016. This way, EFA_1_ and EFA_2_ presented the factor structure of the first version of the PRIME over 2 years of data. Last, we fit EFA_3_, using the 2017 data and all 27 items available for that year. EFA_3_ estimated the factor structure of the second version of the PRIME-Tool with its seven additional items.

The EFAs were fit as follows: First, pairwise correlations were calculated with maximum likelihood estimation and stored in a correlation matrix. Specifically, polychoric correlations were calculated between two ordinal items, tetrachoric correlations between two binary items, biserial correlations between binary and continuous items, and polyserial correlations between ordinal and continuous items [33–35]. Some items that lacked variation in responses caused missing coefficients in the correlation matrices and were not included the EFA: Item #14 (staff are offered training to improve their skills) and item #29 (collects client opinions) were dropped in all EFAs. In addition, item #15 (supervisors have held individual meetings to review staff performance) and item #16 (have established criteria to evaluate staff performance) were dropped in both EFA_2_ and EFA_3_, and item #4 (Has formal goals and priorities for service delivery) was dropped in EFA_3_ for the same reason of lack of variation in responses. Item #34 (has a community member regularly attending staff meetings) and item #7 (burden of target achievement evenly distributed to staff) did not have high enough factor weights to qualify for inclusion in any factors in EFA_2,_ while item #13 (proportion of time facility head spent on managerial activities), item #33 (has a community advisory board that meets regularly), and item #34 (has a community member regularly attending staff meetings) did not have high enough factor weights to qualify for inclusion in any factors in EFA_3_.

Factors were extracted using principal axes factoring. Where needed, we used eigen decomposition and least-squares approximation to obtain positive definite matrices [36]. Once the factors were fit, we retained those with eigenvalues > 1 and implemented both orthogonal varimax and oblique promax rotations [37]. Results from both rotations were overall very similar, but we present results only from the varimax rotation as they were easier to interpret. We retained factors with at least three items with loadings > 0.32. This threshold explains about 10% of common variance with the other items in the factor [37]. This selection of the most influential items improved the conceptual interpretability of each factor.

All statistical analyses were conducted in Stata version 15.1 (StataCorp, LP, College Station, TX).

### Interpretation of EFA factors

We examined factor structure patterns across all three EFA results to guide the development of revised management domains or item groupings. In cases of cross-loadings, we assigned the item to domains where the factor loading was higher or to the domain where it aligns most based on previous theoretical frameworks. To minimize subjectivity in the interpretation and naming of revised management domains given new item groupings, we did a further review of literature and obtained consensus judgment from all co-authors following the recommendation by Tracy (1983) [38, 39]. To interpret the EFA factors, two authors (JU and EM) individually assessed the results and, on the basis of previous theoretical frameworks, proposed revised domain names. Proposed interpretations were cross-checked and revised with all co-authors through group discussion. In cases of discrepancy, a third researcher (AB) participated to resolve the interpretations of the factors.

## Results

The 2016 PRIME-Tool sample included 142 PHC facilities, and the 2017 sample included 148 facilities, of which 137 appeared in both years (Table 1). The five facilities unique to the 2016 sample were all private facilities and of the 11 facilities unique to the 2017 sample, four were private and seven were public. The regional distribution of clinics was similar in both years, with the largest samples in the Ashanti, Western, and Eastern regions, representing about 12 to 17% of the samples each. Roughly half of the facilities were hospitals or polyclinics, another third were health centers and clinics, and the remainder were CHPS. Over 80% of the facilities were public, and over 97% participated in the National Health Insurance system. Facilities sampled in 2016 had on average 51 beds (SD = 63), while facilities in 2017 had an average of 60 beds (SD = 78).

**Table 1:** Characteristics of the primary health care facilities surveyed in Ghana for 2016 and 2017

Characteristic	2016 *N* = 142	2017 *N* = 148
Region, N (%)		
Ashanti	25 (17.6)	24 (16.2)
Brong-Ahafo	13 (9.2)	14 (9.5)
Central	18 (12.7)	17 (11.5)
Eastern	19 (13.4)	19 (12.8)
Greater Accra	12 (8.5)	17 (11.5)
Northern	12 (8.5)	12 (8.1)
Upper East	6 (4.2)	10 (6.8)
Upper West	8 (5.6)	7 (4.7)
Volta	10 (7.0)	10 (6.8)
Western	19 (13.4)	18 (12.2)
Facility type, N (%)		
Hospitals/polyclinics	71 (50.0)	76 (51.4)
Health centers and clinics	48 (33.8)	46 (31.1)
CHPS*	23 (16.2)	26 (17.6)
Managing authority, N (%)		
Public	119 (83.8)	129 (87.2)
Private	23 (16.2)	19 (12.8)
Number of beds, mean (SD)	51 (62.6)	60 (77.8)
Participation in the National Health Insurance System, N (%)	137 (97.2)	145 (98.0)

^a^*CHPS* Community-based Health Planning and Services

Among management indicators, means ranged from 0.24 (item #12 in 2017) to 0.99 (item #14 in both years) on a scale of 0 to 1, with 35 of the 61 total measurements greater than 0.80 (Table 2). Of the 27 items measured in both years, most means were fairly stable over time. Only five items changed by 0.10 points or more on a scale of 0 to 1, with the largest difference in “user fees displayed” (item #12), which dropped by 0.21 points, from 0.45 to 0.24 (Table 2).

**Table 2:** Descriptive analysis and ceiling and floor effects of PRIME-Tool survey items for 2016 and 2017

Item #	Items listed by original hypothesized domain	Variable type^1^	2016 *N* = 142			2017 *N* = 148			Difference in means between years	
			Mean^2^ (Scale: 0 to 1)	% at Floor^3^	% at ceiling^3^	Mean^2^ (Scale: 0 to 1)	% at floor^3^	% at ceiling^3^	Absolute (Scale: 0 to 1)	Percentage points (%)
Target setting										
1	Measures coverage of key population indicators	Y/N	0.92	8.50	**91.50**	0.84	15.54	**84.46**	−0.08	−8.70
2	Has one comprehensive annual budget for running costs	Y/N	0.71	28.87	71.13	0.76	23.60	76.40	+ 0.05	7.04
3	Reports accountability for health outcomes of a group of people	Y/N	0.59	40.80	59.20	0.52	47.97	52.03	−0.07	−11.86
4	Has formal goals and priorities for service delivery	Y/N	4 items not included in 2016			0.95	4.73	**95.27**	–	–
5	Has formal improvement targets to achieve service delivery goals	Y/N				0.45	54.73	45.27	–	–
6	Formal improvement targets for service delivery shared with staff	Y/N				0.89	11.49	**88.51**	–	–
7	Burden of target achievement evenly distributed to facility staff (SD)	Ord.				0.78 (0.19)	0.00	**29.73**	–	–
Operations										
8	Hand washing area with soap and water available (SD)	Ord.	0.95 (0.22)	4.90	**93.66**	0.96 (0.17)	2.70	**93.92**	+ 0.01	1.05
9	Health worker present or on call in the facility 24 h a day	Y/N	0.92	8.45	**91.55**	0.89	10.81	**89.19**	−0.03	−3.26
10	Open every day	Y/N	0.85	14.79	**85.21**	0.90	10.14	**89.86**	+ 0.05	5.88
11	Facility head has received any formal management training	Y/N	0.76	23.94	76.06	0.85	14.86	**85.14**	+ 0.09	11.84
12	User fees displayed (SD)	Ord.	0.45 (0.50)	**54.93**	**18.31**	0.24 (0.38)	**66.89**	**15.54**	−0.21	−46.67
13	Proportion of time facility head spent on managerial activities the previous day (SD)	Cont.	0.43 (0.24)	9.90	1.40	0.39 (0.26)	12.16	3.38	−0.04	−9.30
Human resources										
14	Staff are offered training to improve their skills	Y/N	0.99	1.41	**98.59**	0.99	0.68	**99.32**	0.00	0.00
15	Supervisors have held individual meetings to review staff performance	Y/N	0.95	4.93	**95.07**	0.95	4.73	**95.27**	0.00	0.00
16	Has established criteria to evaluate staff performance	Y/N	0.82	17.61	**82.39**	0.96	4.05	**95.95**	+ 0.14	17.07
17	Has formal, supportive, and continuous supervision system (SD)	Ord.	0.79 (0.29)	4.90	**58.45**	0.89 (0.22)	1.40	**77.03**	+ 0.10	12.66
18	Perceived ability of staff to carry out assignments of daily work (SD)	Ord.	2 items not included in 2016			0.82 (0.21)	2.70	**41.90**	–	–
19	Staff encouraged to share new ideas to management (SD)	Ord.				0.88 (0.14)	0.00	**54.10**	–	–
Monitoring										
20	Maintains books to track revenue and expenditure (SD)	Ord.	0.97 (0.17)	2.80	**54.23**	0.82 (0.26)	2.03	**66.90**	−0.15	−15.46
21	Conducts quality improvement activities	Y/N	0.94	6.34	**93.66**	0.95	4.73	**95.27**	+ 0.01	1.06
22	Held meetings to discuss routine service statistics with staff	Y/N	0.94	5.63	**94.37**	0.95	5.41	**94.59**	+ 0.01	1.06
23	Has mechanism to report new disease outbreaks	Y/N	0.93	7.04	**92.96**	0.97	2.70	**97.30**	+ 0.04	4.30
24	Extent to which data to monitor & improve service delivery is valued (SD)	Ord.	0.88 (0.19)	2.10	**61.97**	0.89 (0.14)	0.00	**59.46**	+ 0.01	1.14
25	Tracks common conditions	Y/N	0.88	11.97	**88.03**	0.91	8.78	**91.22**	+ 0.03	3.41
26	Reports client opinions using any available tool	Y/N	0.54	45.77	54.23	0.54	46.00	54.00	0.00	0.00
27	Regularly receives reports tracking common conditions with results shared with staff (SD)	Ord.	0.41 (0.21)	7.70	2.11	0.40 (0.16)	2.70	1.40	−0.01	−2.44
28	Conducts formal case reviews for quality (SD)	Ord.	Item not included in 2016			0.64 (0.35)	**18.24**	**26.40**	–	–
Community engagement										
29	Collects client opinions using any tool	Y/N	0.95	4.93	**95.07**	0.98	2.03	**97.97**	+ 0.03	3.16
30	Shared information on performance with the community in the past 12 months	Y/N	0.78	21.83	78.17	0.84	16.22	**83.78**	+ 0.08	10.26
31	Patients’ opinions drive change or improvement (SD)	Ord.	0.67 (0.20)	0.70	14.79	0.66 (0.20)	0.68	12.80	−0.01	−1.49
32	Made changes based on client opinion in the last 12 months	Y/N	0.64	35.92	64.08	0.57	43.24	56.76	−0.07	−10.94
33	Has a community advisory board that meets regularly, and facility follows up on board discussions (SD)	Ord.	0.52 (0.49)	**45.07**	**49.30**	0.65 (0.46)	**31.76**	**61.49**	+ 0.13	25.00
34	Has a community member regularly attending staff meetings	Y/N	0.31	69.01	30.99	0.34	66.20	33.80	+ 0.03	9.68

1 Y/N - Binary yes/no variable; Ord. Ordinal variable; Cont. Continuous variable

2 For yes/no variables, the mean is the proportion of facilities that answered yes.

3 Numbers in bold indicate potential ceiling or floor effects (above 80% for yes/no variables and above 15% for ordinal and continuous variables)

Since all measures were scaled between 0 and 1, with 1 signifying better management, these results are generally skewed high, toward better management. As a consequence, 17 of the 27 items included in both versions of the PRIME-Tool showed ceiling effects in both years as indicated in Table 2. Ceiling effects occurred in all five domains: Human resource (all four), Target setting (one of three items), Monitoring (six of eight), Operations (four of six), and Community engagement (two of six). Among the seven items added in 2017, all but one had ceiling effects.

Two items (item #12: user fees displayed and item #33: has a community advisory board that meets regularly) exhibited floor effects as well as ceiling effects in both years, but these were all ordinal variables and had lower thresholds for both floor and ceiling effects than binary items. Only one of the seven items added in 2017 showed floor effects (item #23: has mechanism to report new disease outbreaks).

EFA_1_ and EFA_2_ both suggested that a five-factor solution provided the best fit to the data as it had the fewest factors with eigenvalues > 1 and was the most interpretable of all tested solutions (Table 3). Five factors collectively accounted for 65 and 67% of the variance in the 2016 and 2017 data, respectively. Similarly, EFA_3_ found a six-factor solution explaining 57% of the variance in the 2017 data. With the exclusion of items that lacked variation in responses and items beyond the control of facility management, the final number of items that loaded on at least one factor with a minimum standardized loading of 0.32 was 18 in EFA_1_, 15 in EFA_2_, and 18 in EFA_3_.

**Table 3:** Exploratory Factor Analysis for 2016, 2017, and 2017 with new management indicators

			EFA_1_					EFA_2_					EFA_3_					
			2016 PRIME version 2016 data, *N* = 142 facilities					2016 PRIME version 2017 data, *N* = 148 facilities					2017 PRIME version 2017 data, *N* = 148 facilities					
			Factors					Factors					Factors					
			1	2	3	4	5	1	2	3	4	5	1	2	3	4	5	6
Item #	Original domain	Eigenvalue	2.7	2.7	2.6	2.0	1.9	2.6	2.3	2.2	2.1	1.5	2.6	2.3	2.2	2.0	1.7	1.6
		% Variance accounted for	15%	15%	14%	11%	10%	16%	15%	14%	13%	9%	12%	11%	10%	9%	8%	7%
		Number of items in factor	8	6	7	3	4	5	4	5	7	4	8	6	4	5	3	4
		Item	Factor loadings					Factor loadings					Factor loadings					
24	Monitoring	Extent to which data to monitor & improve service delivery is valued	0.72							0.87			0.45			0.65		
31	Community	Patients’ opinions drive change or improvement	0.65								0.55	−0.38						0.73
17	HR	Has formal, supportive, and continuous supervision system	0.61				−0.40	0.57	0.36					0.68	0.32			
20	Monitoring	Maintains books to track revenue and expenditure	0.58								0.69							0.64
13	Operations	Proportion of time facility head spent on managerial activities the previous day	0.57								0.43		No loadings^2^					
16	HR	Has established criteria to evaluate staff performance	0.48		0.54			Dropped ^1^					Dropped ^1^					
2	Target setting	Has one comprehensive annual budget for running costs	0.48		0.42	0.34		0.36			0.64		0.33				0.52	
11	Operations	Facility head has received any formal management training	0.35		0.62			0.68				0.34		0.37				
26	Monitoring	Reports client opinions using any available tool		0.30	0.67				0.87						0.91			
15	HR	Supervisors have held individual meetings to review staff performance		0.37		0.85		Dropped ^1^					Dropped ^1^					
21	Monitoring	Conducts quality improvement activities		0.55	0.57				0.87	0.43					0.83	0.39		
22	Monitoring	Held meetings to discuss routine service statistics with staff		0.57	0.41			0.85			0.32		0.33	0.74				0.37
27	Monitoring	Regularly receives reports tracking common conditions with results shared with staff		0.83						0.67	0.39		0.84					
23	Monitoring	Has mechanism to report new disease outbreaks		0.94						0.65	0.53		0.85					
32	Community	Made changes based on client opinion in the last 12 months			0.75				0.66	−0.37			−0.31	0.31	0.57			
34	Community	Has a community member regularly attending staff meetings				0.85	0.37	No loadings ^2^					No loadings ^2^					
30	Community	Shared information on performance with the community in the past 6 months					0.70	0.74				0.40	0.31	0.64				
33	Community	Has a community advisory board that meets regularly and facility follows up on board discussions					0.81					0.89	No loadings ^2^					
14	HR	Staff are offered training to improve their skills	Dropped ^1^															
29	Community	Collects client opinions using any tool	Dropped ^1^															
4	Target setting	Has formal goals and priorities for service delivery	7 items not included in 2016 PRIME										Dropped ^1^					
5	Target setting	Has formal improvement targets to achieve goals															0.88	
6	Target setting	Formal improvement targets shared with staff												0.64			0.50	
7	Target setting	Burden of target achievement evenly distributed to staff											No loadings ^2^					
18	HR	Perceived ability of staff to carry out assignments											−0.34			0.68		
19	HR	Staff encouraged to share new ideas to management														0.79		
28	Monitoring	Conducts formal case reviews for quality														0.43		0.55

HR – Human resources

^1^This item was dropped by the EFA because its lack of variation caused missing coefficients in the polychoric correlation matrix.

^2^This item did not load on any of the included factors with a loading of absolute value 0.32 or more

The factor solutions for each EFA suggest that the way items actually correlate in the data did not exactly align with our original conceptual framework or item groupings under the five management domains. Items from different domains loaded together unexpectedly onto factors that may represent new, as-yet-unidentified domains. For instance, the largest factor in EFA_1_ was comprised of eight items with two originally thought to belong to Monitoring, two to the Human resources, two to Operations, and one each to Community engagement and Target setting. In all EFAs, there were several items that consistently exhibited cross loadings or loadings of at least 0.32 in two or more factors such as item #2 (“Has one comprehensive annual budget for running costs”) and item #17 (“Has formal, supportive, continuous supervision system and item”). See Additional file 4 for a summary of results for each item in the PRIME-Tool.

The factor solutions across EFAs do not overlap clearly. For example, the groupings of items with high loadings under factor 1, 2 and, 3 in EFA_1_, were not entirely replicated in EFA_2_ nor EFA_3_. Comparison of the results of EFA_2_ and EFA_3_ showed that the new questions included in the 2017 version of the PRIME-tool altered the factor structure by splitting up items that initially grouped under a factor in EFA_2_. Most notably, the two new items pertaining to Human resources (item #18: “Perceived ability of staff to carry out assignments” and item #19: “Staff encouraged to share new ideas to management”) and the one new item pertaining to Monitoring (item #28: “Conducts formal case reviews for quality”) loaded together strongly with two old items on Monitoring in factor 4 of EFA_3_. This split up a potential “Monitoring” domain comprised only of Monitoring items that loaded together strongly on factor 3 of EFA_2_.

**Fig. 1:**
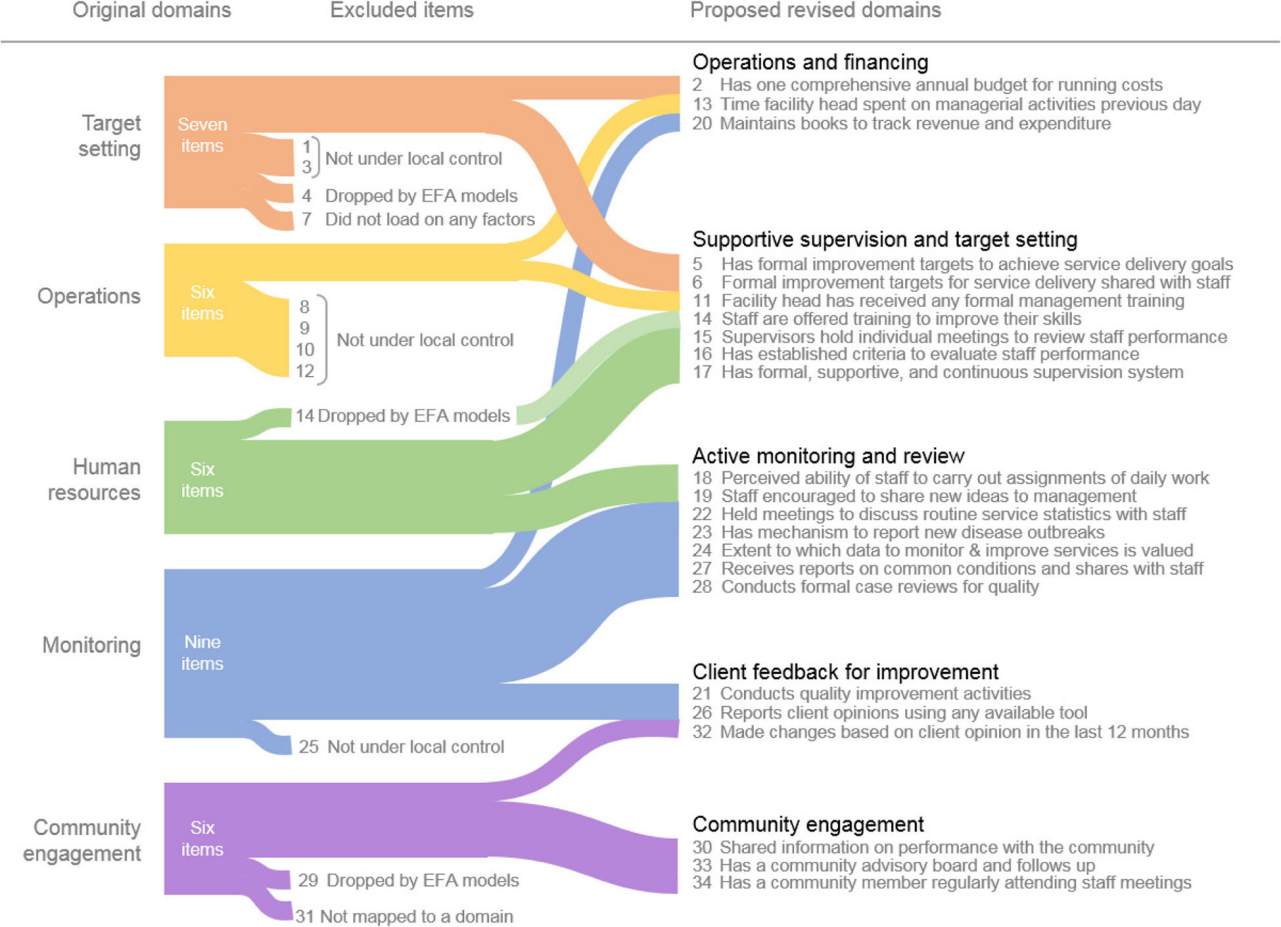
Revised management domains and question groupings

Though the factor structures that emerged were not consistent over each analysis, we found five groupings of items that seemed to consistently load together on the same factors across the three EFAs (Fig. 1). To illustrate, several items under the original “Monitoring” domain frequently loaded together within factors (item #22: Held meetings to discuss routine service statistics with staff, item #23: “Has mechanism to report new disease outbreaks”, and item #27: “Receives and shares reports tracking common conditions with staff”). Through co-author consensus and further literature review, we refined and labeled these revised groupings as the management domains of “Supportive supervision and target setting”, “Active monitoring and review”, “Community engagement, “Client feedback for improvement”, and “Operations and financing.” See Additional file 5 for a detailed description of how the domains were derived and Additional file 6 for revised version of the PRIME-Tool.

## Discussion

There is a distinct lack of valid, reliable, and scalable tools to measure PHC facility management in developing countries. We addressed this gap by developing the PRImary care facility Management Evaluation Tool (PRIME-Tool) - a new tool that can be integrated into routine national surveys in LMICs. Our preliminary validation results shed light on the measurement properties and utilities of the PRIME-Tool. In this section we consider the strengths of the PRIME-Tool, discuss our revised management framework, and identify potential areas for revision.

### Descriptive results and analysis of floor and ceiling effects

Based on our measurements using the PRIME-Tool, this sample of primary care facilities in Ghana seem to be skewed towards good management performance. For both years of data, mean scores of most items were fairly high (> 0.8 on a scale of 0 to 1) and the majority of the items showed stark ceiling effects (Table 2). A possible explanation for these results is that the tool is measuring management performance correctly in these facilities, reflecting the underlying high-performing primary care system in Ghana. Since 1982, Ghana has instituted many key reforms to achieve comprehensive primary health care, with significant investments in reorganizing PHC facilities, building a critical mass of health human resources, and further capacity-building for staff in areas such as facility management [27, 40]. Moreover, the current sample comprised mostly of higher-level facilities, which previous analyses have shown tend to have better management practices [6]. District/primary hospitals are in general better resourced, better staffed, and more likely have established management systems in place than lower level PHC facilities [27, 41].

Alternatively, these results may reflect the presence of some measurement error. Though most means for PRIME-Tool items were fairly stable between 2016 and 2017, there were items with unexpectedly large differences between samples. These differences may appropriately capture genuine changes within the facilities, or they may signal problems with test-retest and inter-rater reliability of the measurement tool. For example, regarding the item #11 “Facility head has received any formal training,” eight facilities changed their response from no to yes and seventeen facilities changed their response from yes to no between 2016 and 2017. It is plausible that the facility head may have attended training between the survey dates or that a new facility head who had not received training was installed. Otherwise, it may be possible that a different facility head may have been interviewed in each year.

### Exploratory factor analysis and revised management domains

Interpreting the EFA results was challenging because the three EFAs did not always present a consistent underlying factor structure based on our original framework, but overlapped in complex ways with each other and with the original domains. Moreover, cross-loadings on more than one factor occurred frequently, a sign that some items may require re-wording. One reason for these cross-loadings are items that ask about two concepts which may apply to more than one management domain. For example, item #27 (“Regularly receives reports tracking common conditions with results shared with staff”) is about tracking health statistics and whether these statistics are shared with staff. Another reason for cross-loadings could be that respondents and enumerators may have had difficulty interpreting questions like item #3 (“Has one comprehensive annual budget for running costs”) where the term “comprehensive” is vague and where past feedback from facility respondents indicated that a comprehensive annual budget is rarely available.

To refine our understanding of PHC facility management in LMICs and capture as much potentially useful information from this exercise as possible, we reconsidered the original five domains of the PRIME-Tool in light of the EFA results. As a result, we propose five revised domains, which echo the original domains but with revised names and different groupings of items (Fig. 1). These revised domains are: “Active monitoring and review”, “Supportive supervision and target setting”, “Operations and financing”, “Community engagement”, and “Client feedback for improvement” (see Additional file 6: The PRIME-Tool).

The process of revisiting our conceptual framework and the resulting revised domains presented an opportunity to advance our understanding of the management of PHC facilities in developing countries. We propose that the revised domains better reflect the management processes that matter most in enabling PHC facilities to accomplish the five core functions of PHC – namely, the 5Cs of first-contact, continuity, comprehensiveness, coordination, and patient-centered care [22, 42].

Comparing the original and revised frameworks, we see a shift in the conceptualization of domains from passive and formal constructs to more active and engaged ones that better align with the nature of PHC. The original “Monitoring” domain has become “Active monitoring and review,” as the items address not only monitoring population indicators, but also internal monitoring of staff and staff empowerment in the use of information from both systems to identify and respond to emerging threats and community needs. Literature supports the concept that improving facility performance through better management involves both monitoring population indicators for planning and directing facility activities, as well as incorporating internal feedback from staff with regards to their ideas on facility targets and their ability to carry out tasks [43]. Internal feedback mechanisms, in particular, have been shown to capture staff self-management and work engagement, reflecting their individual contributions to management performance and facility strategic directions [43–45].

The original “Human resources” domain was concerned with formal structures and systems for managing facility staff that may not be entirely appropriate for community-based operations. The domain was renamed “Supportive supervision and target setting” as it now encompasses items about staff involvement in reaching facility goals. Supportive supervision focuses on improving service delivery and involves a shift in the perception of supervision from traditional checklist-based inspection of performance more related to auditing to an outlook of staff professional development [46–48]. This means that supervision is geared toward ensuring that staff have the support, skills, resources, and the motivation they need to achieve service delivery improvement targets for their community.

“Operations and Financing” and “Community Engagement” remained coherent constructs in our revised framework. However, “Client Feedback for Improvement” emerged as a distinct domain from questions that were originally thought to pertain to Community engagement. While community engagement and people-centered care are close concepts, the PHC literature indicates that the collection and use of individual client feedback to provide care that better meets the needs and expectations of patients is distinct from routine community stakeholder representation in facility management decisions or broader general community engagement [1, 49–51]. Patient engagement mechanisms (e.g. collecting client opinions and using client opinions to drive change in the facility) constitute an important role in building trust between patients and providers as well as feed in to facility quality assurance systems that seek to prevent, detect, and correct problems in the quality of service delivery [52, 53].

### Broad implications of the PRIME-Tool

The PRIME-Tool and its use to measure the management of PHC facilities represents a recognition of the importance of measuring often unseen and undervalued processes that convert resources into coveted health outcomes. Past efforts in PHC have looked primarily into improving inputs and measuring impact in terms of outputs or outcomes - such as in vertical approaches to PHC or disease control [22, 25, 41].

Moreover, this work on the PRIME-Tool shows that it is possible to collect meaningful data on the management of PHC facilities in limited-resource settings. Researchers aiming to develop similar surveys to measure facility management in LMICs may want to take lessons learnt from developing the PRIME-Tool into account: First, it is crucial to ground the tool and its questions on a well-defined framework that focuses on core underlying management functions applicable across facility types – as opposed to specific tasks that may vary in different facility settings. Second, the survey must be as concise as possible in measuring items in the core framework. The tool must be both affordable and feasible to implement given the limited time and bandwidth facility administrators can spare such surveys [54]. As previously discussed, existing evaluation tools for PHC facilities are often focused on measuring the achievement of PHC service delivery tasks and are often unwieldy in length, making implementation in developing countries difficult and costly [15–21].

Last, researchers should think about how the tool can be integrated into country monitoring systems and national surveys to facilitate its long-term sustainability and continued use by the LMIC. As the PRIME-Tool aimed to be as succinct as possible, it was easily incorporated into a routine facility survey in Ghana conducted by the Performance Monitoring and Accountability 2020 (PMA 2020) program [55]. The PRIME-Tool could also be incorporated into other major health facility assessments similar to PMA 2020 such as the Service Provision Assessment of the Demographic and Health Surveys program, the Service Availability and Readiness Assessment of the World Health Organization, and the Service Delivery Indicators launched by the World Bank [19, 20, 56]. On this front, the format, data collection methods, and language of tools to be integrated must be appropriate for enumerators usually employed by monitoring and survey programs. These enumerators are well-trained, but they do not necessarily have extensive knowledge on facility management compared to those employed by the World Management Survey [10].

As PHC has continuously been reaffirmed as a key strategy to achieving the Sustainable Development Goal of universal health coverage, good management of PHC facilities will be a critical in driving the performance of PHC systems in developing countries. Measuring management activities is a key step in identifying gaps in upstream processes that hinder PHC facilities from efficiently and effectively delivering comprehensive and patient-centered care to their communities. The absence of strong measurement tools in the management of PHC facilities, which the PRIME-Tool hopes to address, is thus a significant detriment to the ability of health officials and front-line managers to deliver quality essential quality health care. Data generated from the improved version of the PRIME-Tool may eventually help answer why management practices vary so much across facilities and countries as well as better understand the relationship of management processes with the achievement of PHC functions to ultimately inform system redesigns and other interventions [6, 57].

### Limitations and the PRIME-Tool’s next iterations

Continuing refinement of the PRIME-Tool will address current study and tool limitations through improvements in item construction and further validation studies. Specifically, we plan to modify the PRIME-Tool’s questions by converting yes/no items to ordinal scales of agreement, magnitude, or frequency, to better capture the range of management activities and avoid stark ceiling effects. We will improve the construction of items that cross-loaded heavily or items that did not load on the correct conceptual domain, with the aim of focusing each item on one underlying construct. Underlying constructs will be refined so as to capture only singular core function in PHC facility management. For example, we would like to see a separate factor or domain for “Target setting” which is currently integrated with supportive supervision. Though, it is a part of the supportive supervision process, strategic planning is an important management function that must be distinguished from human resource processes [10, 14].

As we field the PRIME-Tool in other countries, we will further explore the external generalizability of our results in different contexts and facility types. Current EFA findings and floor/ceiling effects found are primarily applicable to this specific sample of facilities in Ghana. At the time of this study’s analysis, the PRIME-Tool had only been implemented in Ghana. Furthermore, though the PMA 2020 sampling design stratifies by private and public sector, it does not stratify by facility level [29]. Consequently, the facility sample under-represents CHPS facilities which work more closely with communities and which may have less rigid management structures [27, 28, 58]. When the PRIME-Tool is fielded in countries with lower- or higher-performing primary care systems and with a more balanced sample of facility types, we will expect the means to respond accordingly. The strong ceiling effects we observed in this study would normally be a sign to revise item wording to improve the ability of the tool to capture variance in management performance. Yet these ceiling effects may not arise in other contexts with fewer health system capacities and resources.

Nevertheless, we believe that our revised framework reasonably captures the management processes essential to the achievement of the 5Cs of PHC. To further this work, our plans for continuous tool development entail more rigorous testing for construct validity, measurement error, and predictive validity. We will conduct a more definitive confirmatory factor analysis to test whether PRIME-Tool items will cluster into the proposed revised management domains in new samples. In addition, we want to address any measurement error in the tool by collecting independent data (i.e., direct observation or administrative data) in parallel to cross-validate the accuracy of responses to items of the PRIME-Tool. Finally, we want to test the ability of items in the PRIME-Tool to predict facility- and patient-level PHC outcomes such as indicators for service availability, family planning, and client satisfaction. We hope to eventually arrive at a more parsimonious tool that is implementable at scale in developing countries and that can produce valid and reliable scores comparable across contexts and predictive of PHC outcomes important to country health staff and officials.

## Conclusions

Measuring PHC facility management in LMICs is complex. The current version of the PRIME-Tool captures a range of important and actionable management information, and we recommend its use by other investigators and practitioners, with no copyright restrictions. Users should keep in mind, however, that the results may exhibit ceiling effects and the factor structure still needs refinement.

Using this study as our starting point, we will continue improving the PRIME-Tool with a goal to arrive at a parsimonious tool measuring clear domains of facility management in PHC relevant to improved quality of service delivery processes and clinical outcomes. At the global level, this study highlights the need for further investments in tools for PHC in low- and middle-income settings. Strong measurement is increasingly necessary as developing nations strive to build vibrant primary health care systems that provide affordable and essential quality health care for all.

## Supplementary information

**Additional file 1.** PRIME-Tool Instrument Development. This file includes a description of how the PRIME-Tool was developed in three stages: (1) adaptation of a management framework, (2) identification and adoption of questions from existing surveys, and (3) revisions after field-testing in Ghana.

**Additional file 2.** PRImary Care Management Evaluation Tool (PRIME-Tool) version 2. This file is the 2017 version of the PRIME-Tool that was assessed in the study for further revisions and improvements.

**Additional file 3.** Map of Ghana administrative regions, districts, and Ashanti enumeration areas. This file includes a map illustrating the geographic and administrative organization of Ghana’s regions, districts, and enumeration areas. All shape files used were uploaded by the Ghana Statistical Service to the Ghana Open Data Initiative website (https://data.gov.gh/about). Data found in this site is publicly available and free for use, modification, or sharing. This figure was generated by authors using GeoDa v1.12.1.139 – a free and open source software developed by Dr. Luc Anselin and his team for spatial analyses.

**Additional file 4:.** Summary of EFA results for each question in PRIME. For each of the three EFAs conducted, the table in this file summarizes on which factor each question in the PRIME-Tool loaded. It also contains a written description detailing which questions loaded together and from which of the original domains they came from.

**Additional file 5:.** Description of Derivation of Revised Management Domains. This document details how the three EFA results and item loadings were interpreted to derive our revised domains.

**Additional file 6:.** PRImary Care Management Evaluation Tool (PRIME-Tool) version 3.This file is the revised and latest version of the PRIME-Tool derived from the results of this study

## Data Availability

The datasets supporting the conclusions of this article may be requested from the PMA 2020 repository (https://www.pma2020.org/request-access-to-datasets) managed and maintained by the Johns Hopkins Bloomberg School of Public Health, Department of Population, Family and Reproductive Health. The Stata codes used for data processing and analyses are available from the corresponding author on reasonable request.

## Abbreviations

CHPS: Community-Based Health Planning and Services
EFA: Exploratory factor analysis
JHSPH: Johns Hopkins School of Public Health
LMICs: Low- and middle-income countries
PHC: Primary health care
PHCPI: Primary Health Care Performance Initiative
PMA 2020: Performance Monitoring and Accountability 2020
PRIME: PRImary care facility Management Evaluation
UHC: Universal health coverage
WMS: World Management Survey
