# Supplementary material for: Preliminary validation of the PRImary care facility Management Evaluation tool (PRIME-Tool), a national facility management survey implemented in Ghana
Source: BMC Health Serv Res. 2019 Dec 5;19:937. doi: 10.1186/s12913-019-4768-8 (PMC6896786; doi:10.1186/s12913-019-4768-8)
Supplement: Supplementary file 1 — Additional file 1. PRIME-Tool Instrument Development. This file includes a description of how the PRIME-Tool was developed in three stages: (1) adaptation of a management framework, (2) identification and adoption of questions from existing surveys, and (3) revisions after field-testing in Ghana. [file 12913_2019_4768_MOESM1_ESM.docx]

**Additional File 1: Description of PRIME-Tool Instrument Development**

The current version of PRIME was developed in three stages: (1) adaptation of a management framework, (2) identification and adoption of questions from existing surveys, and (3) revisions after field-testing in Ghana.

**Selection and Adaptation of a Management Framework**

To anchor all tool development efforts to existing management theory, we first did an extensive review of management frameworks and health facility management in LMICs. We also conducted interviews with 16 experts in facility management, primary health care, health systems, and service delivery, including:

1. Raffaella Sadun, Harvard Business School and World Management Survey
2. Alex Haynes, Ariadne Labs and World Management Survey
3. Mario Macis, Johns Hopkins University, IZA, and National Bureau of Economic Research (NBER)
4. Christophe Rockmore, Service Delivery Indicators (SDI), World Bank
5. Ezequiel Molina, SDI, World Bank
6. Shannon Barkley, PHC Service Delivery and Safety, WHO
7. Erin Sullivan, Harvard Medical School Center for Primary Care
8. Benjamin Loevinsohn, SDI, World Bank
9. Nittita Prasopa-Plazier, Patients for Patient Safety, WHO
10. Giorgio Cometto, Global Health Workforce Alliance, WHO
11. Nuria Toro-Polanco, Integrated People-Centered Health Systems, WHO
12. Kavitha Viswanathan, Health Statistics and Information Systems/Health Facility Harmonization, WHO
13. Shamsuzzoha Syed, Service Delivery and Safety, WHO
14. Hernan Montenegro, Service Delivery and Safety, WHO
15. Herbie Duber, Institute for Health Metrics and Evaluation
16. Erika Linnander, Yale University

The WMS stood out as a particularly important and well-validated framework in measuring facility management across various sectors.^28–33^ The WMS is an innovative research project aimed at measuring the adoption of basic managerial practices. Started in 2001, the initiative has collected in-depth information on management processes across more than 15,000 organizations in manufacturing, retail, education and healthcare in 30 countries. Over the past 5 years, the WMS has conducted more than 1,800 interviews across acute care hospitals in the US, Europe, Asia and South America. Interviewers obtain and conduct interviews that evaluate and score management practices by defining the concept of “good” and “bad” management practices and codifying them from 1 (worst practice) to 5 (best practice) across key management practices used by organizations across different sectors. These practices are grouped into four areas: 1) Operations Management, 2) Performance Monitoring, 3) Target Setting, 4) Talent Management.  (In some WMS formats, a fifth domain: Leadership Management is also added.) Interviews last approximately 90 minutes and are conducted over the phone by extensively trained interviewers. However, given the technical expertise and extensive time commitment needed to conduct, score, and synthesize interviews, the authors determined that using the WMS in its current form would not be feasible.

The authors also met with Dr. Mario Macis of Johns Hopkins University, IZA, and NBER to discuss further options for survey adaptation. Dr. Macis has been involved in the evaluation of a program aimed at implementing SafeCare practices across primary health facilities in Nigeria.^34^ SafeCare is an International Society for Quality in Healthcare (ISQua)-accredited set of standards that are specifically designed for low and middle-income contexts. SafeCare standards cover both clinical services and management functions and are designed to set up a step-wise improvement trajectory for facilities. SafeCare standards were informed by WMS practices but are in a more quantifiable and easily implementable survey format. However, the SafeCare evaluation tool includes more than 800 indicators, making it difficult for a small subset to reliably and fully measure relevant concepts.

Despite these difficulties in adapting World Management Survey questions for facility assessments in LMICs, the authors identified the WMS framework as a valuable tool for structuring a survey module for measuring management of PHC facilities in LMIC. Consequently, we adapted the WMS framework with modification to better apply to PHC facilities in LMICs. Our resulting framework consists of 5 core domains: Target setting, Monitoring, Operations, Human resources, and Community engagement. The first 4 domains were retained from the WMS, and we added a domain for Community engagement as this is essential to the management of PHC facilities.

**Identification and adoption of questions from existing surveys**

Based on our review of the literature and recommendations from consulted experts, authors reviewed the following survey and measurement compilation sources for Facility Management and Organization survey questions and/or frameworks:

- Service Provision Assessment (SPA)^1^
- Service Availability and Readiness Assessment (SARA)^2^
- SafeCare^3^
- Patient Centered Medical Home-Assessment Tool^4^
- Primary Care Assessment Tools (PCAT)^5^
- Primary Care Assessment Survey (PCAS)^6^
- Commonwealth Fund Scorecard^7^
- World Bank and WHO UHC Framework^8^
- WHO Handbook on Monitoring and Evaluation of Human Resources for Health^9^
- Canadian Institute for Health Information, Measuring Organizational Attributes of Primary Health Care Survey^10^
- USAID/Management Sciences for Health Leadership Development Program^11,12^
- Centers for Disease Control and Prevention, Sustainable Management Development Program^13–16^
- WHO Hospital Management Guidelines, 1998^17^
- Mohd-Shamsudin, 2012^18^
- Kitreeawutiwong, 2015^19^
- Munyewende, 2016^20^
- Rowe, 2010^21^
- Ethiopian Hospital Management Initiative^22–27^

Through the group’s in-house expertise in PHC and our understanding of the existing facility management theory, we identified a parsimonious list of questions encompassing key PHC facility management activities for inclusion in the first draft of PRIME.

**First field-testing in Ghana and revisions.**

The first draft of the PRIME was pre-tested in June 2016 in three different types of PHC facilities (public district hospital, private polyclinic, and a CHPS compound) in the peripheral areas of Kumasi, Ghana. To note, these pre-test facilities were excluded from the survey sample. The pre-test aimed to examine the clarity of questions, the acceptability of items to enumerators and facility managers, and the feasibility of integrating the tool into annual country-wide surveys to be conducted through the Performance Monitoring and Accountability 2020 (PMA2020) mobile platform. Field-testing and and enumerator training was led by PMA 2020 staff employed by the Bill & Melinda Gates Institute for Population and Reproductive Health housed in the Johns Hopkins Bloomberg School of Public Health (JHSPH). These activities were supported by staff from the Ariadne Labs Primary Care Team and Kwame Nkrumah University of Science and Technology (KNUST) School of Medicine. Several questions were modified to improve clarity, but all items were acceptable and the survey proved feasible to implement.

The resulting first version of PRIME deployed in Ghana in 2016 had 27 indicators across 5 management domains: 3 in Target setting, 6 in Operations, 4 in Human resources, 8 in Monitoring, and 6 in Community engagement. Based on feedback from enumerators, facility heads, and PMA 2020 staff involved in the first round of data collection, 7 new questions were added to the second version for data collection in 2017. The additional question on formal case reviews in the Monitoring domain is an important indicator of quality assurance systems for patient safety while the 4 new questions in Target setting and 2 in Human resources bolstered the variety of management activities captured by these domains.

The 34 questions were composed of 21 binary indicators (Yes/No or Don’t Know), 12 ordinal indicators and one continuous indicator measuring the proportion of time the PHC facility head spent on managerial activities. Ordinal questions used the following scales: 4 items used a 4-point Likert scale to measure agreement from “strongly disagree” to “strongly agree”, 2 items distinguished between the facility merely reporting the presence of an activity as opposed to being able to show documentation of its conduct, 4 items assigned scores based on the desirability of the type management practice, and 4 used a 5-point Likert scale for activity frequency from “never” to “always.” All scores for response choices are standardized to scale from 0 to 1. A copy of the current version of the PRIME with the score assignments for each choice is available in Additional file 2.

**References**

1. The DHS Program - Service Provision Assessments (SPA). https://dhsprogram.com/What-We-Do/Survey-Types/SPA.cfm (accessed Jan 21, 2018).
2. WHO | Service Availability and Readiness Assessment (SARA). WHO 2015.
3. Safecare: Basic HealthCare Standards. http://www.safe-care.org/ (accessed Jan 21, 2018).
4. PATIENT-CENTERED MEDICAL HOME ASSESSMENT (PCMH-A) Introduction To The PCMH-A. .
5. Primary Care Assessment Tools. https://www.jhsph.edu/research/centers-and-institutes/johns-hopkins-primary-care-policy-center/pca_tools.html (accessed Jan 21, 2018).
6. Safran DG, Kosinski M, Tarlov AR, et al. The Primary Care Assessment Survey: Tests of Data Quality and Measurement Performance. Med Care 1998; 36: 728–39.
7. Health System Scorecards - The Commonwealth Fund. http://www.commonwealthfund.org/publications/health-system-scorecards (accessed Jan 21, 2018).
8. Monitoring progress towards universal health coverage at country and global levels. 2014.
9. Handbook on Monitoring and Evaluation Human Resources for Health: with special applications for low-and middle-income countries Handbook on Monitoring and Evaluation of Human Resources for Health. 2009.
10. Measuring Organizational Attributes of Primary Health Care Survey. https://www.cihi.ca/sites/default/files/info_phc_organize_en.pdf (accessed Jan 21, 2018).
11. Kwamie A, van Dijk H, Agyepong IA. Advancing the application of systems thinking in health: realist evaluation of the Leadership Development Programme for district manager decision-making in Ghana. Heal Res policy Syst 2014; 12: 29.
12. Seims LRK, Alegre JC, Murei L, et al. Strengthening management and leadership practices to increase health-service delivery in Kenya: an evidence-based approach. Hum Resour Health 2012; 10: 25.
13. Centers for Disease Control. Sustainable Management Development Program: Two Decades of Improving Health Outcomes Through Strong Leadership and Management. 2002 DOI:10.1146/annurev.ecolsys.33.010802.150507.
14. Umble KE, Brooks J, Lowman A, et al. Management training in Vietnam’s National Tuberculosis Program: an impact evaluation. Int J Tuberc Lung Dis 2009; 13: 238–46.
15. Sucaldito NL, Tayag EA, Roces MCR, Malison MD, Robie BD, Howze EH. The Philippines Field Management Training Program (FMTP): strengthening management capacity in a decentralized public health system. Int J Public Health 2014; 59: 897–903.
16. McEwan E, Conway MJ, Bull DL, Malison MD. Developing Public Health Management Training Capacity in Nicaragua. Am J Public Health 2001; 91: 1586–8.
17. Conn CP, Jenkins P, Touray SO. Strengthening health management: Experience of district health teams in The Gambia. Health Policy Plan 1996; 11: 64–71.
18. Mohd-Shamsudin F, Chuttipattana N. Determinants of managerial competencies for primary care managers in Southern Thailand. J Health Organ Manag 2012; 26: 258–80.
19. Kitreerawutiwong K, Sriruecha C, Laohasiriwong W. Development of the competency scale for primary care managers in Thailand: Scale development. BMC Fam Pract 2015; 16: 174.
20. Munyewende PO, Levin J, Rispel LC. An evaluation of the competencies of primary health care clinic nursing managers in two South African provinces. Glob Health Action 2016; 9: 32486.
21. Rowe LA, Brillant SB, Cleveland E, et al. Building capacity in health facility management: guiding principles for skills transfer in Liberia. Hum Resour Health 2010; 8: 5.
22. McNatt Z, Linnander E, Endeshaw A, Tatek D, Conteh D, Bradley EH. A national system for monitoring the performance of hospitals in Ethiopia. Bull World Health Organ 2015; 93: 719–26.
23. Hartwig K, Pashman J, Cherlin E, et al. Hospital Management in the context of health sector reform: a planning model in Ethiopia. Int J Health Plann Manage 2008; 23: 203–18.
24. Kebede S, Abebe Y, Wolde M, Bekele B, Mantopoulos J, Bradley EH. Educating leaders in hospital management: a new model in Sub-Saharan Africa. Int J Qual Health Care 2010; 22: 39–43.
25. Kebede S, Mantopoulos J, Ramanadhan S, et al. Educating leaders in hospital management: A pre-post study in Ethiopian hospitals. Glob Public Health 2012; 7: 164–74.
26. Linnander E, McNatt Z, Sipsma H, et al. Use of a national collaborative to improve hospital quality in a low-income setting. Int Health 2015; 8: 148–53.
27. Bradley E, Hartwig K a, Rowe L a, et al. Hospital quality improvement in Ethiopia : a partnership – mentoring model. Int J Qual Heal Care 2008; 20: 392–9.
28. Tsai TC, Jha AK, Gawande AA, Huckman RS, Bloom N, Sadun R. Hospital board and management practices are strongly related to hospital performance on clinical quality metrics. Health Aff 2015; 34: 1304–11.
29. Bloom N, Sadun R, Van Reenen J. Does Management Matter in Healthcare? London School of Economics Working Paper, 2013.
30. Bloom N, Propper C, Seiler S, Reenen J Van. CEP Discussion Paper No 983 May 2010 ( Revised November 2014 ) The Impact of Competition on Management Quality : Evidence from Public Hospitals. 2013; 2010.
31. McConnell KJ, Hoffman KA, Quanbeck A, McCarty D. Management practices in substance abuse treatment programs. J Subst Abuse Treat 2009; 37: 79–89.
32. Lemos R, Scur D. Could Poor Management be Holding Back Development? Describing practices in the public and private sectors in India. 2012; : 53.
33. McConnell KJ, Lindrooth RC, Wholey DR, Maddox TM, Bloom N. Management practices and the quality of care in cardiac units. JAMA Intern Med 2013; 173: 684–92.
34. Dunsch FA, Evans DK, Macis M, Giorgi G De, Loevinsohn B, Odutolu O. Management, Supervision, and Health Care: A Field Experiment. 2017.
