# Supplementary material for: Preliminary validation of the PRImary care facility Management Evaluation tool (PRIME-Tool), a national facility management survey implemented in Ghana
Source: BMC Health Serv Res. 2019 Dec 5;19:937. doi: 10.1186/s12913-019-4768-8 (PMC6896786; doi:10.1186/s12913-019-4768-8)
Supplement: Supplementary file 3 — Additional file 3. Map of Ghana administrative regions, districts, and Ashanti enumeration areas. This file includes a map illustrating the geographic and administrative organization of Ghana’s regions, districts, and enumeration areas. All shape files used were uploaded by the Ghana Statistical Service to the Ghana Open Data Initiative website (https://data.gov.gh/about). Data found in this site is publicly available and free for use, modification, or sharing. This figure was generated by authors using GeoDa v1.12.1.139 – a free and open source software developed by Dr. Luc Anselin and his team for spatial analyses. [file 12913_2019_4768_MOESM3_ESM.docx]

**Additional File 3: Map of Ghana administrative regions, districts, and Ashanti enumeration areas**

**
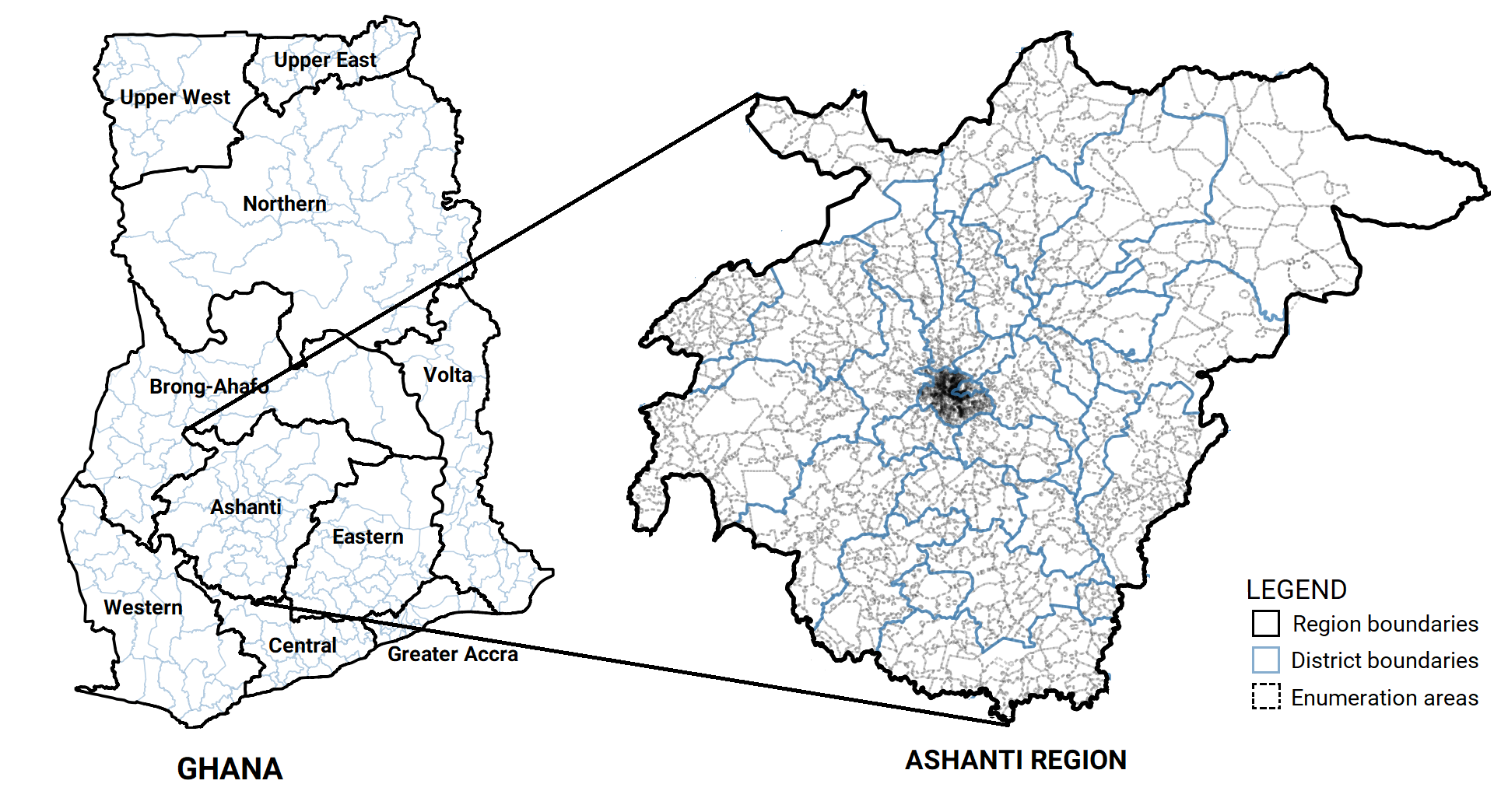
**

**Source**: All shape files used were uploaded by the Ghana Statistical Service to the Ghana Open Data Initiative website (<https://data.gov.gh/about>). Data found in this site is publicly available and free for use, modification, or sharing.

<https://data.gov.gh/dataset/shapefiles-all-regions-ghana-2010-10-regions>

<https://data.gov.gh/dataset/shapefiles-all-districts-ghana-170-districts>

<https://data.gov.gh/dataset/shapefiles-enumeration-areas-ashanti-region>

This figure was generated by authors using GeoDa v1.12.1.139 – a free and open source software developed by Dr. Luc Anselin and his team for spatial analyses.
