# Supplementary material for: Preliminary validation of the PRImary care facility Management Evaluation tool (PRIME-Tool), a national facility management survey implemented in Ghana
Source: BMC Health Serv Res. 2019 Dec 5;19:937. doi: 10.1186/s12913-019-4768-8 (PMC6896786; doi:10.1186/s12913-019-4768-8)
Supplement: Supplementary file 4 — Additional file 4:. Summary of EFA results for each question in PRIME. For each of the three EFAs conducted, the table in this file summarizes on which factor each question in the PRIME-Tool loaded. It also contains a written description detailing which questions loaded together and from which of the original domains they came from. [file 12913_2019_4768_MOESM4_ESM.docx]

**Additional file 4: Summary of EFA results for each question in PRIME**

| Original domain | Item  # | Item | EFA_1_  2016 questions  2016 data | EFA_2_  2016 questions  2017 data | EFA_3_  2017 questions  2017 data | Recommended final domain |
| --- | --- | --- | --- | --- | --- | --- |
| Target setting | 1 | Measures coverage of key population indicators | Dropped: Not under local control | | |  |
| Target setting | 2 | Has one comprehensive annual budget for running costs | Factors 1,3,4 | Factors 1,4 | Factors 1,5 | Operations and Financing |
| Target setting | 3 | Reports accountability for health outcomes of a group of people | Dropped: Not under local control | | |  |
| Target setting | 4 | Has formal goals and priorities for service delivery | Not in 2016 PRIME | | Dropped by  EFA model |  |
| Target setting | 5 | Has formal improvement targets to achieve service del goals | Not in 2016 PRIME | | Factor 5 | Supportive Supervision and Target Setting |
| Target setting | 6 | Formal improvement targets for service delivery shared w staff | Not in 2016 PRIME | | Factors 2,5 | Supportive Supervision and Target Setting |
| Target setting | 7 | Burden of target achievement evenly distributed to facility staff | Not in 2016 PRIME | | No factors |  |
| Operations | 8 | Hand washing area with soap and water available | Dropped: Ceiling effects and not a management activity | | |  |
| Operations | 9 | Health worker present or on call in the facility 24 hours a day | Dropped: Not under local control | | |  |
| Operations | 10 | Open every day | Dropped: Not under local control | | |  |
| Operations | 11 | Facility head has received any formal management training | Factors 1,3 | Factors 1,5 | Factor 2 | Supportive Supervision and Target Setting |
| Operations | 12 | User fees displayed | Dropped: Not under local control | | |  |
| Operations | 13 | Proportion of time facility head spent on managerial activities | Factor 1 | Factor 4 | No factors | Operations and Financing |
| Human resources | 14 | Staff are offered training to improve their skills | Dropped by EFA model | | | Supportive Supervision and Target Setting |
| Human resources | 15 | Supervisors have held individual mtgs to review staff perfce | Factors 2,4 | Dropped by EFA model | | Supportive Supervision and Target Setting |
| Human resources | 16 | Has established criteria to evaluate staff performance | Factors 1,3 | Dropped by EFA model | | Supportive Supervision and Target Setting |
| Human resources | 17 | Has formal, supportive, and continuous supervision system | Factors 1,5 | Factors 1,2 | Factor 2 | Supportive Supervision and Target Setting |
| Human resources | 18 | Perceived ability of staff to carry out assignments of daily work | Not in 2016 PRIME | | Factors 1,4 | Active Monitoring and Review |
| Human resources | 19 | Staff encouraged to share new ideas to management | Not in 2016 PRIME | | Factor 4 | Active Monitoring and Review |
| Monitoring | 20 | Maintains books to track revenue and expenditure | Factor 1 | Factor 4 | Factor 6 | Operations and Financing |
| Monitoring | 21 | Conducts quality improvement activities | Factors 2,3 | Factors 2,3 | Factors 3,4 | Client Feedback for Improvement |
| Monitoring | 22 | Held meetings to discuss routine service statistics with staff | Factor 2,3 | Factor 1,4 | Factors 1,2,6 | Active Monitoring and Review |
| Monitoring | 23 | Has mechanism to report new disease outbreaks | Factor 2 | Factor 3,4 | Factor 1 | Active Monitoring and Review |
| Monitoring | 24 | Extent to which data to monitor & improve service del is valued | Factor 1 | Factor 3 | Factors 1, 4 | Active Monitoring and Review |
| Monitoring | 25 | Tracks common conditions | Dropped: Not under local control | | |  |
| Monitoring | 26 | Reports client opinions using any available tool | Factor 3 | Factor 2 | Factor 3 | Client Feedback for Improvement |
| Monitoring | 27 | Regularly receives reports tracking common conditions with results shared with staff | Factor 2 | Factor 3,4 | Factor 1 | Active Monitoring and Review |
| Monitoring | 28 | Conducts formal case reviews for quality | Not in 2016 PRIME | | Factors 4,6 | Active Monitoring and Review |
| Community eng | 29 | Collects client opinions using any tool | Dropped by EFA model | | |  |
| Community eng | 30 | Shared information on perf with the community in the past 12 m | Factor 5 | Factors 1,5 | Factors 2 | Community Engagement |
| Community eng | 31 | Patients’ opinions drive change or improvement | Factor 1 | Factors 4,5 | Factor 6 |  |
| Community eng | 32 | Made changes based on client opinion in the last 12 months | Factor 3 | Factor 2,3 | Factor 3 | Client Feedback for Improvement |
| Community eng | 33 | Has a community advisory board that meets reg and … | Factor 5 | Factor 5 | No factors | Community Engagement |
| Community eng | 34 | Has a community member regularly attending staff meetings | Factors 4,5 | No factors | No factors | Community Engagement |

EFA_1_ yielded a five factor solution that collectively accounted for 65% of the variance in the 2016 data. The largest factor in EFA_1_ accounted for 15% of the variance, and was comprised of eight items with factor loadings above 0.32 (item #24: extent data to monitor and improve service delivery is valued, item #31: patient opinion drives change or improvement, item #17: has formal, supportive, continuous supervision system, item #20: maintains books to track revenues and expenses, item #13: proportion of time facility head spent on managerial activities, item #16: have established criteria to evaluate staff performance, item #2: has one comprehensive annual budget for running costs, and item #11: facility head has received formal management training, as listed in order of factor loading in Table 3). Of these eight, two were originally hypothesized to be in the Monitoring domain, two in the Human Resources domain, two in Operations, and one each in Community engagement and Target setting. This suggests that the original hypothesized domains for these items were not fully consistent with the way the items actually correlated in the 2016 data.

The second factor of EFA_1_ included five items originally from the Monitoring domain (item #21: conducts quality improvement activities, item #22: held meetings to discuss routine service statistics with staff, item #23: has mechanism to report new disease outbreaks, item #26: reports client opinion, and item #27: receives and shares reports tracking common conditions with staff) as well as a fifth from Human resources (item #15: supervisors have held individual meetings to review staff performance). This represented a better match between the hypothesized domain of Monitoring and this factor, but the three other items hypothesized to be in the Monitoring domain (item #24: extent data to monitor and improve service delivery is valued, item #20: maintains books to track revenues and expenses, and item #28: conducts formal case reviews for quality) loaded on to other factors. The loadings in the remaining three factors in EFA_1_ show similar patterns, with limited overlap with the original domains, frequent cross loadings, and unexpected groupings into new, as-yet-unidentified domains.

EFA_2_ also found a five-factor solution, which accounted for 67% of the 2017 variance. Note that although these factors are numbered 1 to 5, they are not necessarily capturing the same qualities as factors 1 to 5 from EFA_1_. In fact, the EFA_2_ factors do not clearly overlap with either the original hypothesized domains or the domains in EFA_1_. Its first factor explains 16% of the 2017 variance and includes five items, one from each of the five original hypothesized domains. These five items also loaded in various ways onto all five factors found in EFA_1_.

EFA_3_ included data from several new items on the 2017 version of the PRIME-Tool that were expected to load into one of the original five hypothesized domains. EFA_3_ found a six factor solution explaining 57% of the 2017 variance, and again these factors did not overlap clearly with the original domains or the factors of EFA_1_ and EFA_2_.
