# Supplementary material for: Preliminary validation of the PRImary care facility Management Evaluation tool (PRIME-Tool), a national facility management survey implemented in Ghana
Source: BMC Health Serv Res. 2019 Dec 5;19:937. doi: 10.1186/s12913-019-4768-8 (PMC6896786; doi:10.1186/s12913-019-4768-8)
Supplement: Supplementary file 5 — Additional file 5:. Description of Derivation of Revised Management Domains. This document details how the three EFA results and item loadings were interpreted to derive our revised domains. [file 12913_2019_4768_MOESM5_ESM.docx]

**Additional file 5: Description of Derivation of Revised Management Domains**

**Active Monitoring and Review**

This domain largely reflects the original “Monitoring” domain. Many of the seven items in this revised domain (item #18: perceived ability of staff to carry out assignments of daily work, item #19: staff encouraged to share new ideas to management, item #22: held meetings to discuss routine service statistics with staff, item #23: has mechanism to report new disease outbreaks, item #24: extent to which data to monitor and improve service delivery is valued, item #27: regularly receives reports tracking common conditions with results shared with staff, and item #28: conducts formal case reviews for quality) appeared together in factor 2 in EFA_1_, factors 3 and 4 in EFA_2_, and factors 1 and 4 in EFA_3_. All items except item #19 cross-loaded onto other factors in at least one EFA, but we ultimately grouped them in this domain based on the magnitude of the various factor loadings, theory from the literature, and co-author consensus. The name was revised to “Active monitoring and review,” as the items address not only monitoring population indicators, but also internal monitoring of staff and the use of information from both systems.

**Supportive Supervision and Target Setting**

This revised domain is based on the original “Human Resources” domain. Of its seven items (item #5: has formal improvement targets for service delivery goals, item #6: formal improvement targets shared with staff, item #11: facility head has received formal management training, item #14: staff are offered training to improve their skills, item #15: supervisors have held individual meetings to review staff performance, item #16: established criteria to evaluate staff performance, and item #17: has formal, supportive, continuous supervision system), four originated in Human Resources. This revised domain was derived from factor 1 in EFA_1_, factor 1 in EFA_2,_ and factors 2 and 5 in EFA_3_. Although item #14 (staff are offered training to improve their skills) was dropped by the EFA models due to lack of variation, we included it in this domain due to its central conceptual importance in the management literature, as well as the possibility of more variation in other contexts.

**Operations and Financing**

The “Operations and financing” domain is comprised of items about the budget tracking and personnel management. It includes item #2 (has comprehensive annual budget for running costs), item #13 (proportion of time facility head spent on managerial activities), and item #20 (maintains books to track revenues and expenses), derived from factor 1 of EFA_1_ and factor 4 of EFA_2_. Item #2 was originally in Target Setting and item #20 was originally in Monitoring, but they both address budgeting and finance. Their clustering with item #13, which was originally in Operations, suggests that together they may capture an underlying management quality having to do with both operations and financing. Though item #31 (client opinion drives change) actually clustered with these operations and financing items, we decided to exclude it from this domain. Conceptually, item #31 was meant to be a measure of people-centered care, and its grouping with operations and financing items may indicate problems with item construction.

**Community Engagement**

This domain includes item #30 (shared information with community in past 12 months), item #33 (has community advisory board that meets regularly), and item #34 (has a community member regular attending staff meetings) that constitutes the “Community engagement” domain which loaded together in factor 5 in EFA_1_ and factor 5 in EFA_2_. These items all originated in “Community Engagement,” so the name of the revised domain is unchanged.

**Client Feedback for Improvement**

This domain was formed from item #21 (conducts quality improvement activities), item #26 (reports client opinions), and item #32 (patient’s opinions drive change or improvement) as they loaded together on factor 3 in EFA_1_, factor 2 in EFA_2_, and factor 3 in EFA_3._
