# Supplementary material for: Preliminary validation of the PRImary care facility Management Evaluation tool (PRIME-Tool), a national facility management survey implemented in Ghana
Source: BMC Health Serv Res. 2019 Dec 5;19:937. doi: 10.1186/s12913-019-4768-8 (PMC6896786; doi:10.1186/s12913-019-4768-8)
Supplement: Supplementary file 6 — Additional file 6:. PRImary Care Management Evaluation Tool (PRIME-Tool) version 3.This file is the revised and latest version of the PRIME-Tool derived from the results of this study [file 12913_2019_4768_MOESM6_ESM.docx]

**Additional file 6:**

**PRImary Care Management Evaluation (PRIME) v.3**

**Active Monitoring and Review**

| 1 | **Perceived ability of staff to carry out assignments of daily work:** How much to you agree with this statement:  “Staff in this facility have the ability to determine how to carry out the assignments of their daily work.” | | | | | | | | | | |
| --- | --- | --- | --- | --- | --- | --- | --- | --- | --- | --- | --- |
|  | Never  0 | Rarely  0.25 | | | Sometimes  0.5 | | | Most of the times  0.75 | | | Always  1 |
| 2 | **Staff encouraged to share new ideas to management:** How much do you agree with this statement:  “Staff are encouraged to bring forward new ideas.” | | | | | | | | | | |
|  | Never  0 | Rarely  0.25 | | | Sometimes  0.5 | | | Most of the times  0.75 | | | Always  1 |
| 3 | **Held meetings to discuss routine service statistics with staff:** In the past 12 months, have there been any meetings where routinely collected service statistics or clinical audit data are discussed with staff? | | | | | | | | | | |
|  | No/ Don’t know  0 | | | | | Yes  1 | | | | | |
| 4 | **Has mechanism to report new disease outbreaks:** Do you have a mechanism to collect and report new disease outbreaks? | | | | | | | | | | |
|  | No/ Don’t know  0 | | | | | Yes  1 | | | | | |
| 5 | **Extent to which data to monitor & improve service delivery is valued: H**ow much do you agree or disagree with the following statement: The use of data to monitor and improve service delivery is highly valued in this facility. | | | | | | | | | | |
|  | Strongly disagree  0 | | Disagree  0.25 | | Neither  0.5 | | Agree  0.75 | | | Strongly agree  1 | |
| 6 | **Regularly receives reports tracking common conditions with results shared with staff:**  Are the results of the health conditions and outcomes collected and shared with facility staff through any means?  Tools can include: displayed in the facility (chalkboard, poster, noticeboard), staff meetings, on individual basis as requested, not collected or shared? | | | | | | | | | | |
|  | No tools/no collection  0 | | | One tool used  0.25 | | Two tools used  0.5 | | | All three tools used  0.75 | | |
| 7 | **Conducts formal case reviews for quality:** Does this facility routinely carry out formal case reviews, that is, where individual patient management is reviewed for quality and potential for improved case management?  How frequently are formal case reviews carried out? | | | | | | | | | | |
|  | Never/No specified timing  0 | | Less than quarterly  0.25 | | At least quarterly  0.5 | | At least monthly  0.75 | | | At least weekly  1 | |

**Client feedback and improvement**

| 8 | | **Conducts quality improvement activities:** Does this facility conduct any quality improvement activities? | | |
| --- | --- | --- | --- | --- |
|  |  | No/ Don’t know  0 | | Yes  1 |
| 9 | | **Reports client opinions using any available tool:** Is there a mechanism for the facility to collect patient opinions and feedback? | | |
|  |  | No/ Don’t know  0 | | Yes (any tool)  1 |
| 10 | **Made changes based on client opinion in the last six months:** In the past 12 months have any changes been made in the program as a result of client opinion? | | | |
|  | No/ Don’t know  0 | | Yes  1 | |

**Operations and Financing**

| 11 | **Has comprehensive annual budget for running costs:** Does your facility have one comprehensive annual budget for running costs?  By running costs, I mean all of the costs of operating this facility, including paying staff, building maintenance, and purchasing of supplies, equipment, medicines, and utilities. | | | | |
| --- | --- | --- | --- | --- | --- |
|  | No/ Don’t know  0 | | Yes  1 | | |
| 12 | **Proportion of time facility head spent on managerial activities the previous day** | | | | |
|  | a. How many total hours did you work [yesterday] at this facility? | | | Hours | |
|  | During this day, how much time did you devote to each of these activities: | | | | |
|  | b. Overseeing patient flow  (e.g., patient admissions, triage, transfers, and discharges) | | | Hours | |
|  | c. Supervising medical staff  (e.g., meeting with staff, providing feedback, checking absenteeism) | | | Hours | |
|  | d. Managing operational budgets  (e.g., tracking revenue, submitting claims, paying bills) | | | Hours | |
|  | e. Verifying or ensuring the availability of drugs and equipment  (e.g., taking inventory, placing orders, etc.) | | | Hours | |
|  | f. Treating patients yourself  (e.g., providing consultations) | | | Hours | |
|  | g. Managing relationships with staff, community, facility committee, donors, and government | | | Hours | |
|  | g. Other | | | Hours | |
|  | **% Time spent on managerial activities** | | | (c. + d. + e. + f.)  a. | |
| 13 | **Maintains books to track revenue and expenditure:** Does your facility maintain books to track revenue and expenditures? May I see them? | | | | |
|  | None  0 | Reported only  0.5 | | | Observed  1 |

**Supportive supervision and target setting**

| 14 | **Has formal improvement targets to achieve service delivery goals:** Has your facility formally established improvement targets to achieve these goals? | | | |
| --- | --- | --- | --- | --- |
|  | No/ Don’t know  0 | | Yes  1 | |
| 15 | **Formal improvement targets for service delivery shared with staff:** Are these targets shared with staff? | | | |
|  | No/ Don’t know  0 | | Yes  1 | |
| 16 | **Facility head has received any formal management training:** Have you ever received formal training in management of a health facility? | | | |
|  | No/ Don’t know  0 | | Yes  1 | |
| 17 | **Staff are offered training to improve their skills:** Are staff in the facility offered trainings to improve their skills? | | | |
|  | No/ Don’t know  0 | | | Yes  1 |
| 18 | **Supervisors have held individual meetings to review staff performance:** In the past 12 months, have supervisors at your facility held individual meetings with staff to review their performance? | | | |
|  | No/ Don’t know  0 | | | Yes  1 |
| 19 | **Has established criteria to evaluate staff performance:** Do you have a set of established criteria your facility uses to evaluate staff performance? | | | |
|  | No/ Don’t know  0 | | | Yes  1 |
| 20 | **Has formal, supportive, and continuous supervision system:** What is the main method of supervision in place in your facility? | | | |
|  | 0 | No method of supervision | | |
|  | 0.33 | Supervision is only available if requested by staff. | | |
|  | 0.33 | Supervision consists of negative feedback when performance is poor. | | |
|  | 0.66 | Formal supervision process with regular pre-arranged supervision meetings. | | |
|  | 1 | Supervision is supportive and continuous. | | |

**Community Engagement**

| 21 | **Shared information on performance with the community in the past 12 months:** In the past 12 months, has this facility shared information on its performance with the community it serves? | | | |
| --- | --- | --- | --- | --- |
|  | No/ Don’t know  0 | | Yes  1 | |
| 22 | **Has a community advisory board that meets regularly and follows up:** Does this facility have a community advisory board or community management committee that meets regularly?  Has the facility taken any follow-up action on the basis of discussions had during the last meetings? | | | |
|  | No CAB  0 | Has CAB, no follow-up  0.5 | | Has CAB, follows-up  1 |
| 23 | **Has a community member regularly attending staff meetings:** Is there a community member who regularly attends staff meetings? | | | |
|  | No/ Don’t know  0 | | Yes  1 | |
